# SPATS2 is positively activated by long noncoding RNA SNHG5 via regulating DNMT3a expression to promote hepatocellular carcinoma progression

**DOI:** 10.1371/journal.pone.0262262

**Published:** 2022-01-25

**Authors:** Jia Yan, Qing Yu Huang, Ya Jun Huang, Chang Shan Wang, Peng Xia Liu

**Affiliations:** College of Life Science, Inner Mongolia University, Hohhot, China; Chung Shan Medical University, TAIWAN

## Abstract

Hepatocellular carcinoma (HCC) is one of the most prevalent malignant tumors with high mortality worldwide. *Spermatogenesis-associated serine-rich* 2 (*SPATS2*) could be a novel diagnostic and prognostic biomarker in HCC. However, the regulatory mechanism of SPATS2 in HCC requires further elucidation. Therefore, the study’s objective was to investigate this process in HCC. In this study, we found that *SPATS2* is significantly upregulated in HepG2 cells to promote cell growth and migration. *SPATS2* is the target transcript of lncRNA *SNHG5*. SPATS2 positively affects the proliferation and migration of HepG2 cells caused by the higher expression of *SNHG5*. Mechanistically, we identified that the elevated of *SPATS2* was attributed to *SNHG5* related hypomethylation of *SPATS2*. *SNHG5* reduced the expression of *DNMT3a* to suppress the methylation level of *SPATS2*. Taken together, our results uncover a novel epigenetic regulatory mechanism of lncRNA SNHG5-DNMT3a axis-related *SPATS2* expression underlying HCC progression. This may serve as a novel prognostic marker and a promising therapeutic target for the treatment of HCC.

## Introduction

Hepatocellular carcinoma (HCC) is the sixth most common cancer and one of the greatest threats to human health worldwide [1]. The incidence of HCC is increasing rapidly in China [2]. Despite the rapid developments in therapy, the overall survival rate of patients is still low due to its complex pathogenesis, the heterogeneity of the cancer cells, the high recurrence rate, and resistance to chemotherapy [3]. Thus, reliable biomarkers are crucial for the early diagnosis and development of novel strategies for the effective treatment of patients with HCC.

SPATS2 is dysregulated in a few types of cancers such as HCC, squamous cell carcinoma, and colorectal carcinoma (CRC) [4, 5]. In liver cancer, it is highly expressed and could predict poor prognosis [6]. A recent study indicated that SPATS2 promotes hepatocellular carcinoma progression by regulating the cell cycle [7]. Additionally, SPATS2 serves as a target transcript of the small nucleolar RNA hostgene5 SNHG5 in colorectal cancer cells [8]. SNHG5 expression was found to be closely related to the tumor range, lymph node metastasis, distant metastasis and prognosis of cancer [9]. Previous pieces of evidence indicated that SNHG5 promotes human HCC progression by regulating the miR-26a-5p/GSK3β [10], miR-23c/HMGB2 [11], and miR-363-3p/RNF38 axis [12]. Although an increasing number of studies have elucidated the biological function of SNHG5 in liver cancer, the function and regulatory mechanism of SNHG5 in SPATS2 expression has not been reported.

To date, noncoding RNA regulation and DNA methylation have been the best-investigated epigenetic alterations in HCC. Statistical data show that genetic alterations frequently occur in epigenetic modifiers, which account for approximately 20–50% of HCC cases [13]. DNMTs (DNA methyltransferases) and Tets (ten-eleven translocation hydroxylases) family proteins regulate targeted transcriptional gene repression tightly. DNMT1, DNMT3a, and DNMT3b are significantly elevated in HCC compared with nonneoplastic liver tissues. TET family proteins were generally downregulated, but TET1 was expressed to a certain extent in HCC cells. Numerous studies have revealed that lncRNAs may epigenetically suppress miRNA expression via DNMT-mediated DNA methylation.

A recent study found that lncRNAs act as ceRNAs to affect the expression of DNMT3s through miRNAs [14]. Moreover, in addition to acting as molecular sponges for microRNAs [15, 16], lncRNAs can also inhibit target gene expression through the recruitment of DNMTs to their promoters, such as lncRNA IRAIN/DNMT3s/VEGFA [17], lncRNA MCM3AP-AS1/DNMT1/DNMT3(A/B)/NPY1R [18], and lncRNA ROIT/DNMT3a/Nkx6.1 [19]. Here, to achieve the goal of elucidating the regulatory mechanism of SPATS2 in HCC, we raise the question of whether SNHG5 could regulate SPATS2 expression through DNA demethylation.

In this study, we first found that SNHG5 directly targets SPATS2 and results in hypomethylation of the promoter of SPATS2 by inhibiting the expression of DNMT3a. Our data suggest that the SNHG5-DNMT3a-SPATS2 axis exerts a vital function in HCC development and might be a promising therapeutic target for HCC.

## Materials and methods

### Cell culture

Human HCC cell lines (HepG2, Huh7, MHCC-97H, MHCC-97L), and the immortalized human hepatic cell line HL-7702 (L02) were purchased from the Type Culture Collection of the Chinese Academy of Sciences (Shanghai, China). All cells were cultured in a 6-well plate with DMEM (Dulbecco’s Modified Eagle Medium) (Gibco, USA) supplemented with 10% FBS (Fetal Bovine Serum) (Gibco, USA), 100 μg/mL streptomycin and 100 U/mL penicillin (Sigma, USA) at 37°C in a 5% CO_2_ atmosphere saturated with water. Microscopic observation shows epithelial-like morphology and adherent growth. The procedures used in this study adhere to the tenets of the Declaration of Helsinki.

### Plasmid construction and transfection

The overexpression plasmid and its negative control (NC), small interfering RNA (siRNA) and its negative control (siNC) were synthesized by Gene Pharma Company (Shanghai, China). All the oligonucleotides or vectors were transfected into HepG2 cells using Lipofectamine 2000 transfection reagent (Invitrogen, CA, USA) according to the manufacturer’s protocol at approximately 50–70% cell confluence. Then cells were harvested for subsequent analysis.

### RNA isolation and quantitative RT-PCR

Total RNA was extracted using TRIzol reagent (Invitrogen, CA, USA) according to the manufacturer’s protocol. cDNA was obtained according to the protocol of the Prime ScriptTM RT Master Mix Kit (Takara, Japan), and qRT-PCR was performed using SYBR Premix Ex Taq^™^II (Takara). The relative gene expression level was calculated using the 2−ΔΔCt method. Each independent experiment was replicated at least three times.

### Cell viability assay (MTT assay)

Cell proliferation was measured by the MTT assay (Roche Diagnostics, Switzerland). After transfection for 24, 48, 72, and 96 h, cells were digested and collected, subsequently cultured in medium with 0.5 mg/ml MTS and kept in the dark for 4 h. Cell viability was detected at 490 nm by an EnSpire Multimode Plate Reader (PerkinElmer, Germany). Each independent experiment was replicated at least three times.

### Colony formation assay

The transfected cells in the logarithmic growth phase were collected and resuspended after digestion with trypsin. Then, the appropriate amount of cell suspension was added to 6.0 cm culture dishes. The cells were grown in a humidified incubator (5% CO_2_, 37°C). After routine incubation for 14 days, the colonies were washed with PBS (phosphate-buffered saline), fixed with 10% formaldehyde, stained with 1% crystal violet for 30 min and the number of colonies formed was counted. Each independent experiment was replicated at least three times.

### Cell cycle assay

The cells were harvested and fixed in 70% ethanol for 2 h. After washing the cells twice with precooled PBS, the cells were then treated with propidium iodide (PI)/RNase staining buffer (BD Biosciences Pharmingen, USA) according to the manufacturer’s protocol. Then, the samples were immediately analyzed by flow cytometry (BD Canto II, USA). The results were analyzed by FlowJo 10 software (BD Canto II, USA).

### Cell apoptosis assay

The cells were harvested and then treated with FITC Annexin V apoptosis detection kit I (BD Biosciences Pharmingen, USA) according to the manufacturer’s protocol. The samples were immediately analyzed by flow cytometry (BD Canto II, USA). The results were analyzed by FlowJo 10 software (BD Canto II, USA).

### Cell migration assay

Cells were cultured in 6-well plates. After transfection for 24 h, the cells reached 90%~100% confluence. The cells were scratched with 100 μl pipette tips, and 3 lines were drawn in parallel. Subsequently, the cells were washed out twice with PBS buffer and cultured in serum-free DMEM at 37°C with 5% CO_2_. After 24 h of wound formation, the wound size was measured and photographed. The migration of cells was analyzed using ImageJ 1.48 software. The wound healing rate was calculated as follows: wound healing rate = [(scratch width at 0 h)-scratch width at 24 h]/ (scratch width at 0 h)] ×100%. Each independent experiment was repeated at least three times.

### Western blot analysis

Total proteins were extracted from cells after transfection for 48 h using RIPA buffer with proteinase (10% NP-40, 10% sodium deoxycholate, 100 mM NaCl, 20 mM Tris-HCl pH 7.4, 100 mM EDTA), and then protein concentrations were detected using a BCA protein assay (Thermo Fisher Scientific, Inc.) according to the manufacturer’s protocol. Each sample was separated by 10% SDS-PAGE and transferred onto PVDF membranes. After blocking with 5% nonfat milk for 1 h at room temperature, the membranes were incubated with the indicated antibodies overnight at 4°C. Then, the membranes were incubated with HRP-conjugated secondary antibodies for 1 h at room temperature after washing three times with TBST solution. Signals were visualized using ECL substrates (Millipore). Each independent experiment was replicated at least three times.

### Luciferase reporter assay

To detect the binding of SNHG5 and the 3′-UTR of SPATS2. Luciferase reporter construct containing regions of SPATS2 was cloned into the pMIR-REPORT luciferase vector (RiboBio, Guangzhou, China). The assays were performed 48 h after transfection of the indicated constructs into 4 × 10^4^ 293T cells per well (three wells per sample) seeded in 96-well plates. The cells were obtained, and the luciferase results were determined using the Dual Glo Luciferase Assay System (Promega, Wisconsin, USA) and GloMax Multi Detection System (Promega, Wisconsin, USA). Renilla luciferase activity was used as the control. Each independent experiment was replicated at least three times.

### Pyrosequencing analysis of bisulfite converted DNA

Genomic DNA was extracted using a genomic DNA purification kit (Qiagen Company, Hilden, Germany). Bisulfite modification of DNA was performed by the EZ DNA Modification Kit (Zymo Company, New York, USA) according to the manufacturer’s protocol. Bisulfite-treated DNA was PCR amplified using SPAYS2 specific primers. Primers were designed using Pyrosequencing Assay Design Software v2.0 (Qiagen, Germany). Subsequently, the PCR product (10 uL) and magnetic beads (3 uL) were pipetted into the PyroMark Q48 disk wells and loaded on the Q48 instrument (Qiagen, Germany) for PSQ. Amplification was conducted according to guidelines suggested by Pyrosequencing Assay Design Software. The methylation level was analyzed using the Q48 Autoprep software 2.4.2 on CpG analysis mode and visualized as sequence-specific programs.

### Patient data analysis

The TCGA Liver cancer (LIHC) transcriptional data and clinical data were downloaded. The term SPATS2 and SNHG5 was used to search the cBioPortal database (http://www.cbioportal.org/). In DNA methylation research, we evaluated the ex-pression and methylation level of SPATS2 in liver cancer by UALCAN (http://ualcan.path.uab.edu/). The SPATS2 methylation status in liver cell were assessed using the MethBank database.

### Statistical analysis

All experiments were independently performed at least three times with biological repeats, and the results are expressed as the mean ± SD. One-way analysis of variance was applied for the comparison among multiple groups, and Student’s t-test was used for comparing the data between two groups. Differences were defined as statistically significant for *P*-values <0.05. All statistical analyses were performed in SPSS 21.0 software. Correlations between two groups were analyzed using Pearson’s correlation coefficient analysis.

## Results

### SPATS2 is highly expressed and associated with poor prognosis of HCC patients

**Fig 1:**
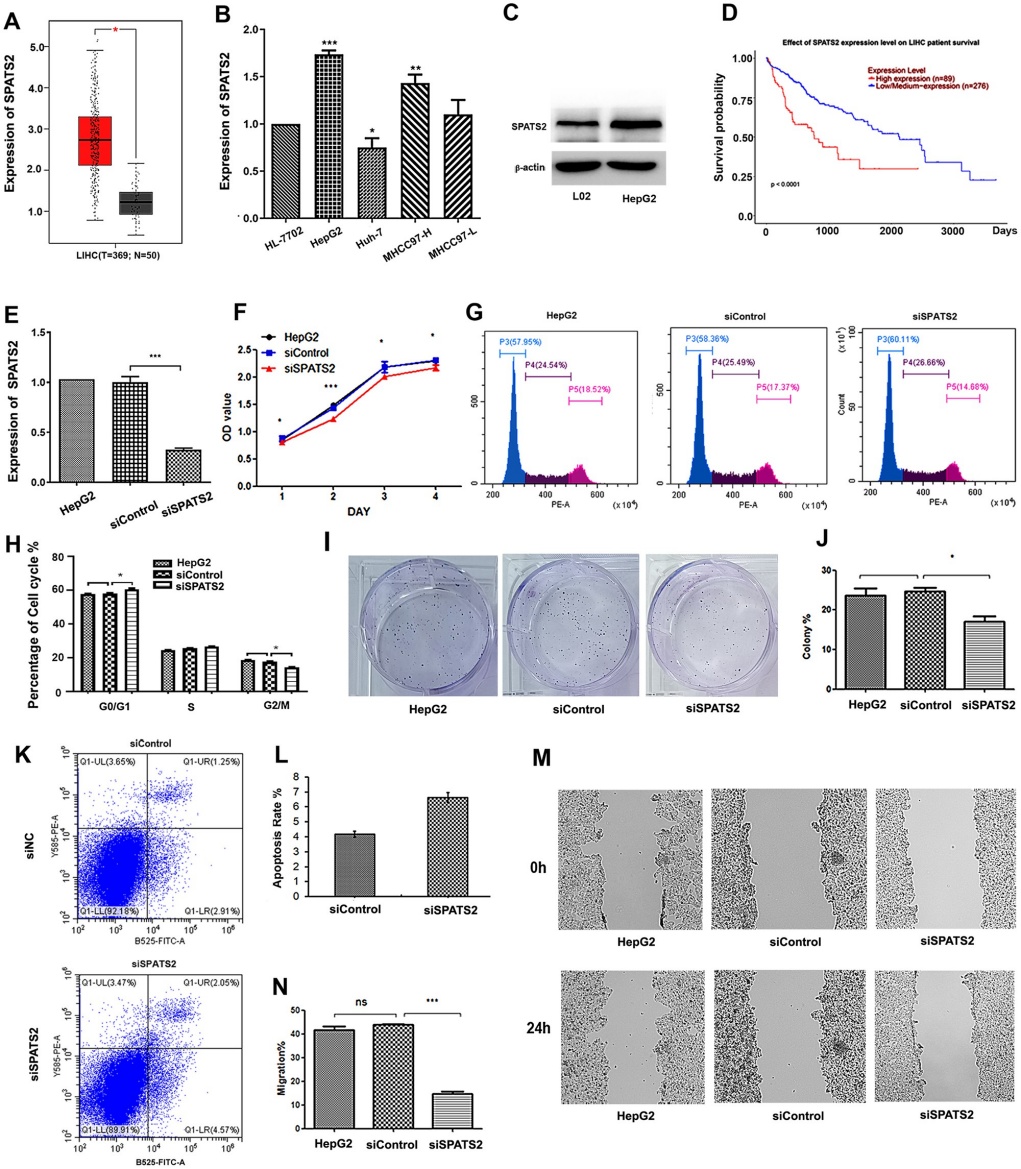
Knockdown of *SPATS2* inhibits the malignant phenotypes of HepG2 cells. (A) Expression of SPATS2 in HCC tissues and adjacent normal liver tissues. (B) mRNA levels of *SPATS2* in different HCC cell lines. (C) Protein levels of SPATS2 in HepG2 and normal cells. (D) Survival curves were analyzed by Kaplan-Meier survival analysis. (E) Expression of *SPATS2* in siSPATS2-transfected HepG2 cells (F) MTT assays detected that the proliferation of *SPATS2* downregulated HepG2 cells. (G, H) FACS analysis showed that downregulation of *SPATS2* represses the cell cycle in HepG2 cells. (I, J) SPATS2 knockdown induced a decrease in the clonogenic survival ability of HepG2 cells. (K, L) FACS analysis showed that downregulation of SPATS2 induces apoptosis of HepG2 cells. (M, N) Wound healing assays in siSPATS2 HCC cells. The experiment was repeated three times, and a representative blot image is shown. *p < 0.05, **p < 0.01, ***p < 0.01.

In order to explore the function of SPATS2 in HCC. *SPATS2* expression patterns in patient samples were derived from TCGA database, *SPATS2* was more highly expressed in human primary liver cancer tissue (n = 369) than in normal human liver tissue (n = 50) (Fig 1A). The qRT-PCR results showed that *SPATS2* was significantly higher in HCC HepG2 cells than in L02 human normal liver cells (Fig 1B). Western blot results also showed that SPATS2 accumulated in HepG2 cells (Fig 1C). Moreover, Kaplan-Meier analysis (log-rank test) demonstrated that the elevated *SPATS2* was related to poor overall survival of liver cancer patients (p<0.0001) (Fig 1D).

### SPATS2 is critical for cell proliferation and migration in HCC cells

To explore the roles of SPATS2, a specific siRNA was introduced into HepG2 cells to reduce endogenous *SPATS2* expression. The endogenous *SPATS2* level was significantly knocked down in HepG2 cells by siRNA (Fig 1E). We evaluated the impact of *SPATS2* gene knockdown on cell proliferation, apoptosis and the cell cycle in HepG2 cells (Fig 1F–1L). The MTT assay showed that the proliferation of HepG2 cells was significantly inhibited (Fig 1F). In detail, knockdown of *SPATS2* caused fewer cells in S phase and more cells in the G1 arrest phase (Fig 1G and 1H). The colony formation ability was inhibited (Fig 1I and 1J). The apoptosis ratio of HepG2 cells was significantly increased by *SPATS2* knockdown, as detected by flow cytometry analysis (Fig 1K and 1L). Moreover, wound healing assays showed that the metastatic ability was reduced, indicating that *SPATS2* promotes the migration of liver cancer cells (Fig 1M and 1N).

### SPATS2 is a key downstream target of SNHG5 in HCC

**Fig 2:**
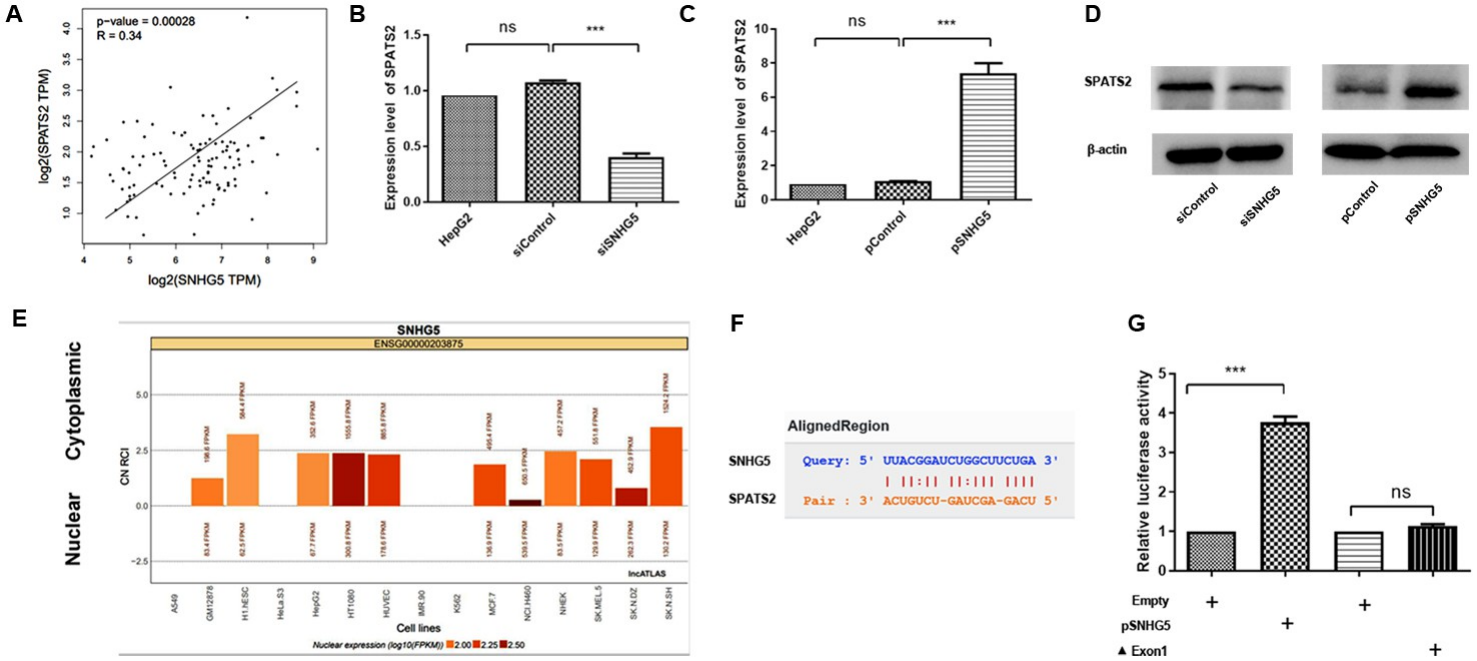
SPATS2 is downstream target of SNHG5 in HepG2 cells. (A) Pearson’s correlation analysis of the relationship between SNHG5 and SPATS2 expression in HCC tissues. (B-D) SPATS2 expression in SNHG5 overexpression- or downregulation-transfected HepG2 cells. (E) Cell location of SNHG5. (F) Sequence alignment of SPATS2 with the putative binding sites in the wild-type regions of SNHG5. (G) A luciferase reporter assay verified the targeted binding effect between the SPATS2 3′UTR and SNHG5. The experiment was repeated three times, and a representative blot image is shown. **p < 0.01, ***p < 0.001.

TCGA database analysis revealed that upregulated *SPATS2* was correlated with higher expression of *SNHG5* in liver cancer (Fig 2A). To verify whether *SNHG5* promotes the expression of *SPATS2* in human liver cancer, we performed *SNHG5* gene knockdown and overexpression experiments in HepG2 cells. The results showed that *SPATS2* mRNA levels were significantly reduced in siSNHG5 HepG2 cells and increased in SNHG5-overexpressing HepG2 cells (Fig 2B and 2C). More importantly, the protein level of SPATS2 was also significantly reduced in siSNHG5 HepG2 cells and increased in SNHG5-overexpressing HepG2 cells (Fig 2D).

SPATS2 was predicted to be a cytoplasmic RNA-binding protein. To further explore the regulatory mechanism between SPATS2 and SNHG5 in HepG2 cells, we first used the lncATLAS website (http://lncatlas.crg.eu/) to predict the subcellular localization of SNHG5. The results revealed that SNHG5 was mainly localized in the cytoplasm (Fig 2E). The RNA interaction ENCORI database result showed that SPATS2 is a potential target transcript of SNHG5 (Fig 2F). We then assessed the binding of *SNHG5* to the 3′-UTR of *SPATS2* using a luciferase reporter system in 293T cells. The overexpression of *SNHG5* significantly increased the luciferase activity of the *SPATS2* 3′ UTR reporter construct compared with the *SNHG5* mutant lacking exon 1 (Fig 2G). SPATS2 is a downstream target of SNHG5 in HepG2 cells.

### Overexpression of SNHG5 promotes HCC cell proliferation and migration in HepG2 cells

**Fig 3:**
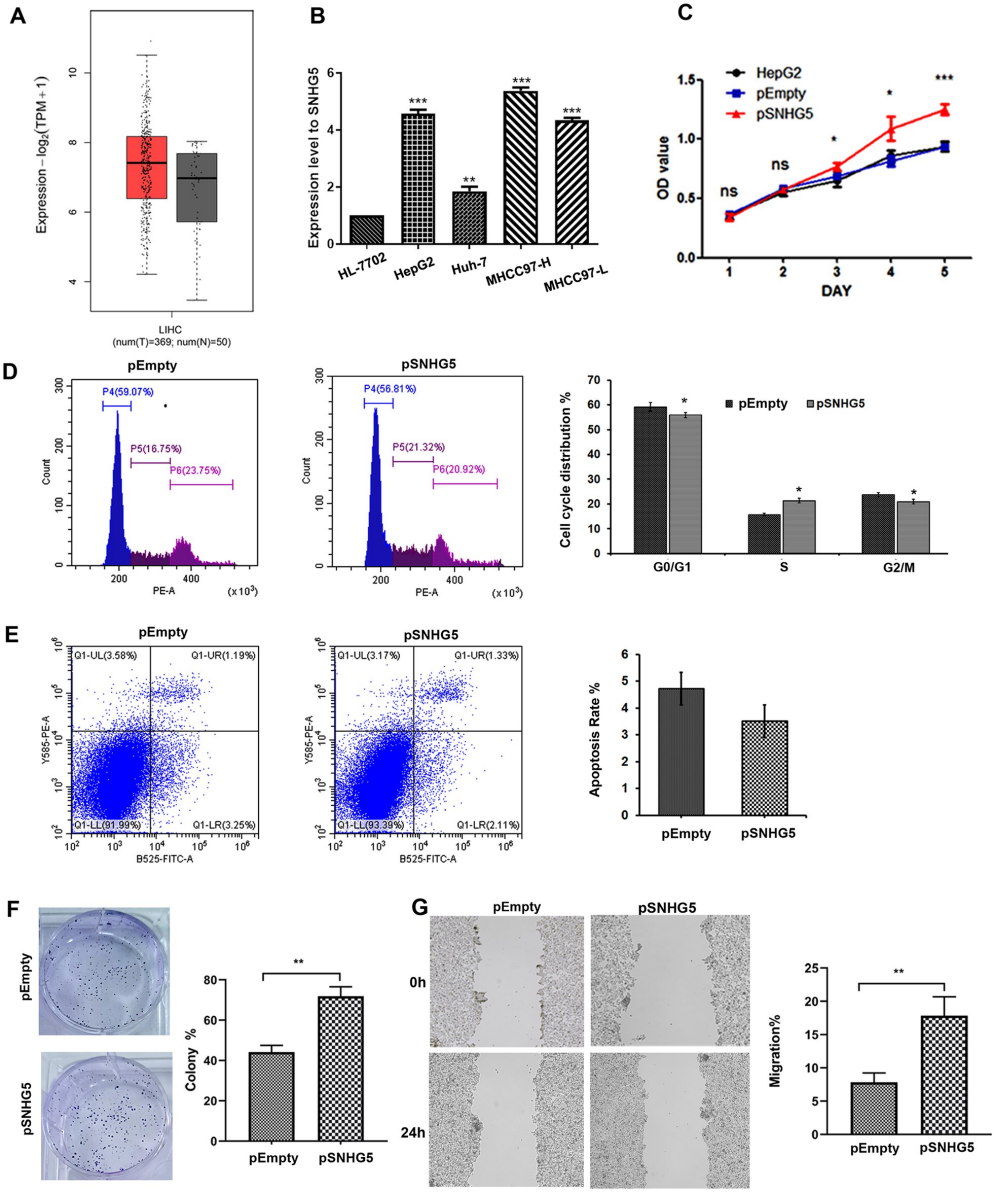
Overexpression of *SNHG5* promotes proliferation and migration in HCC line. (A) The SNHG5 expression in HCC tissues and normal tissues. (B) Differential expression patterns of SNHG5 in different HCC cell lines. (C, D) Overexpression of SNHG5 promoted cell proliferation and the cell cycle; (E) repressed cell apoptosis, and increased the clonogenic survival ability (F) of HepG2 cells. (G) Wound healing assays in SNHG5 overexpressed HepG2 cells. *P < 0.05, **P < 0.01, **P < 0.001.

We further analyzed SNHG5 function in HCC. TCGA database showed that SNHG5 was elevated in liver cancer tissue samples (Fig 3A). Moreover, SNHG5 was up-regulated in different HCC cell lines (Fig 3B). Furthermore, overexpression of SNHG5 in HepG2 cells promoted cell proliferation by facilitating cell cycle progression (Fig 3C and 3D) and repressed cell apoptosis (Fig 3E), which was further supported by colony formation assays (Fig 3F). In addition, SNHG5 overexpression significantly promoted the migration ability of HepG2 cells as detected by the wound-healing assay (Fig 3G). Thus, SNHG5 also exerts an oncogenic role in HCC cells by promoting cell proliferation, inhibiting cell apoptosis and enhancing the migration ability.

### SNHG5 promotes HCC tumorigenesis by promoting the expression of SPATS2

**Fig 4:**
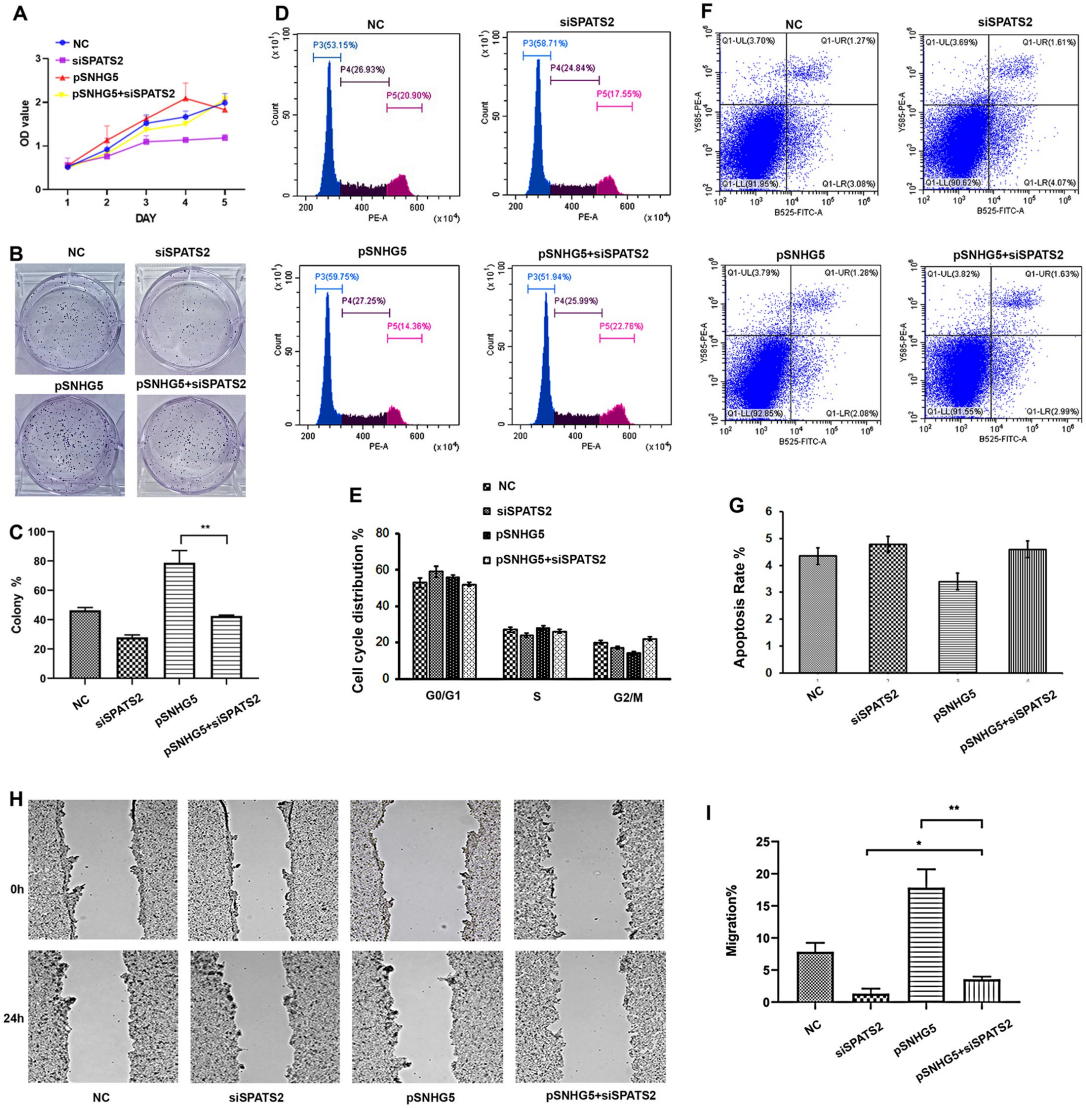
Overexpression of SNHG5 promotes proliferation and migration in the SPATS2 knockdown HepG2 cell line. (A-C) Overexpression of SNHG5 promoted cell proliferation (A) increased the clonogenic survival ability (B, C), abnormal cell cycle (D-E), repressed cell apoptosis (F, G) in SPATS2 knockdown HepG2 cells. (H, I) Wound healing assays after SNHG5 was overexpressed in SPATS2 knockdown HepG2 cells. *P < 0.05, **P < 0.01, **P < 0.001.

To elucidate whether SNHG5 promotes HCC tumorigenesis by promoting the expression of SPATS2, siSPATS2 was introduced into SNHG5-overexpressing HepG2 cells to downregulate *SPATS2* levels. Indeed, in the presence of high levels of *SNHG5* expression, knockdown of *SPATS2* led to a significant reduction in cell viability (Fig 4A–4I). The results showed that siSPATS2 transfection could attenuate the promotion effect of SNHG5 on cell growth. The MTT assay showed that the proliferation of HepG2 cells was significantly inhibited (Fig 4A). Meanwhile, the colony formation ability was also abolished (Fig 4B and 4C). The flow cytometry analysis showed that cell cycle progression and repressed cell apoptosis were recovered after downregulating SPATS2 in SNHG5 overexpressed HepG2 cells (Fig 4D–4G). In addition, wound healing assays showed that downregulated SPATS2 reduced migration ability of SNHG5 overexpressed HepG2 cell lines (Fig 4H and 4I).

### DNA methylation involves the expression regulation of SPATS2 in HepG2 cells

**Fig 5:**
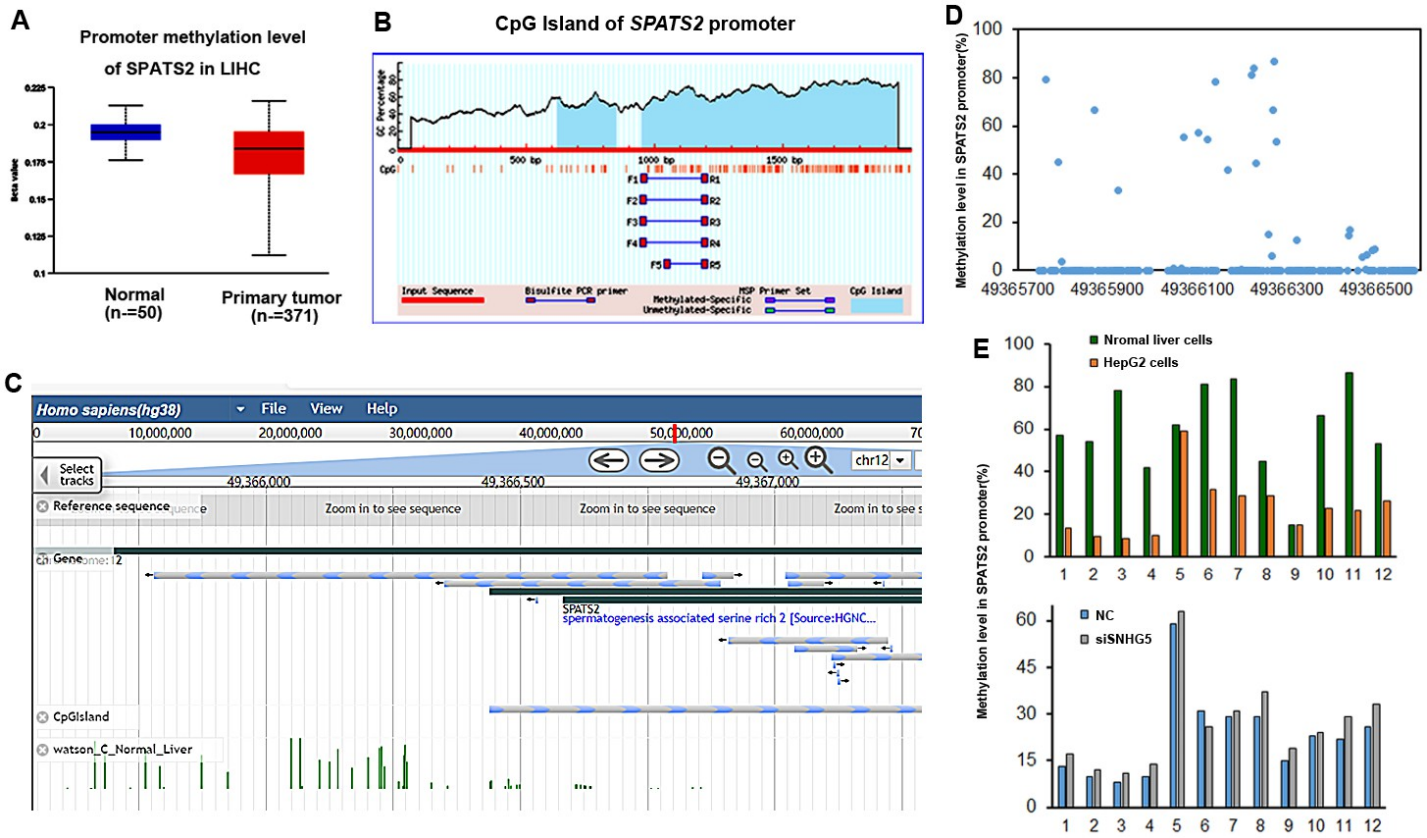
Expression of SPATS2 is associated with promoter methylation. (A) Hypomethylation of SPATS2 promoter in HCC tissues. (B) CpG Island of *SPATS2* promoter region on the MethPrimer website. (C, D) Methylation level of SPATS2 promoter in normal liver cell. (E) Methylation level of SPATS2 in HepG2 and SNHG5 knockdown HepG2 cells.

In order to study the regulatory mechanism by how *SNHG5* regulates *SPATS2* expression in HCC, the correlation between gene expression and methylation level in *SPATS2* was analyzed. TCGA results showed that the methylation level of *SPATS2* promoter region was decreased in liver cancer tissue (Fig 5A). Moreover, CpG islands were embedded in *SPATS2* gene promoter (Fig 5B). The MethBank Database also showed that CpG island was located in *SPATS2* promoter region (Fig 5C). Furthermore, we assessed the methylation levels of different CpG dinucleotides located at 49366102 to 49366300 regions in the *SPATS2* promoter. The average methylation level in this region is 60.4% (Fig 5D). We then adopted pyrosequencing assay to assess its methylation status in HepG2 cells, whose results showed that the methylation frequency of these CpG dinucleotides in *SPATS2* promoter was significantly decreased in HepG2 cells. On the contrary, knockdown of SNHG5 led to higher methylation levels of these CpG dinucleotides in *SPATS2* promoter. (Fig 5E). Taken together, SNHG5 could epigenetically induced SPATS2 expression by keeping the SPATS2 promoter hypomethylated in HCC.

### SNHG5 is associated with DNMT3a to decrease the DNA methylation levels of SPATS2

**Fig 6:**
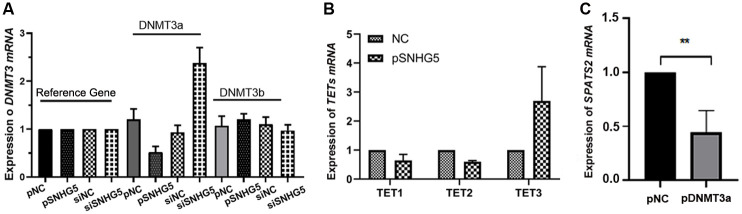
SNHG5 inhibits DNMT3a expression to up-regulate SPATS2. (A, B) RT-qPCR detected the relative expression of DNMT3a and TETs (TET1, TET2, TET3) in SNHG5-overexpression and downregulation HepG2 cells. (C) DNMT3a overexpression induces downregulation of SPATS2. The experiment was repeated three times, and a representative blot image is shown. *p < 0.05, **p < 0.01.

Based on the results that SNHG5 was mainly localized in the cytoplasm predicted in lncATLAS website and binds to the 3’ UTR of SPATS2. We speculated that SNHG5 may participate in the methylation status of SPATS2, indirectly. To further investigate how SNHG5 promotes the expression of SPATS2 through DNA hypomethylation, we examined the mRNA expression of the DNMT3 and TETs in HepG2 cells. DNMT3a was significantly inhibited by SNHG5 overexpression, while siSNHG5 dismissed this inhibition in HepG2 cells (Fig 6A). In addition, TET1 and TET2 were decreased, and TET3 was increased by overexpression of SNHG5 in HepG2 cells, but its increase was not significant (Fig 6B). To further confirm that the hypomethylation of SPATS2 is caused by low expression of DNMT3a, we overexpressed DNMT3a in HepG2 cells. As expected, the mRNA level of SPATS2 was decreased in HepG2 cells when DNMT3a was overexpressed (Fig 6C). In summary, the SNHG5-DNMT3a axis regulates SPATS2 expression in HepG2 cells.

## Discussion

SPATS2 has been reported to contribute to the tumorigenesis of multiple malignancies. Recent research has found that knockdown of *SPATS2* represses tumor growth and metastasis *in vivo* [7]. In this study, we discovered that the expression of SPATS2 was associated with SNHG5 in HepG2 cells. Moreover, its expression was affected by DNA methylases. Recent discoveries have also identified lncRNAs as new important players in DNA methylation regulation. For example, lncRNA IRAIN inhibits VEGFA expression or MCM3AP-AS1 upregulates NPY1R through recruitment of DNMT1-, DNMT3a-, and DNMT3b-mediated methylation [15, 16]. In here, we found that SNHG5 is mainly localized in the cytoplasm and binds to the 3’ UTR of SPATS2. we speculated that SNHG5 may participate in the methylation status of SPATS2, indirectly.

Many previous studies have elaborated on the functions of lncRNA SNHG5 in detail [9]. However, all evidence has focused on SNHG5 acting as a sponge for microRNAs in HCC. In the present study, we aimed to shed light on the potential mechanism by which lncRNA SNHG5 regulates the expression of SPATS2 to affect HCC tumorigenesis and development. Our results demonstrated that SNHG5 could affect the methylation of *SPATS2*. In addition, we found that SNHG5 inhibits the expression of DNMT3a in HepG2 cells. This result may be related to the previous conclusion that lncRNA acts as a ceRNA to affect the expression of DNMT3a. For example, lncRNA HIF1A-AS3 affects the expression of DNMT3a through downregulation of miR-129-5 [20]; the lncRNA-SNHG7/miR-29b/DNMT3a axis affects the activation, autophagy and proliferation of hepatic stellate cells in liver fibrosis [21]. Here, there may be similar miRNA-related regulatory mechanisms that result in decreased DNMT3a expression. Some miRNAs and the detailed regulatory mechanism need to be verified in our future work.

Currently, different therapeutic approaches based on both molecular and cellular therapies have been developed. Evidence from recent research indicate that targeting epigenetic regulators can control HCC progression [22]. In addition, SNHG5 has been reported to be upregulated in sorafenib-resistant HepG2 cell [23]. Our results indicate that SNHG5 related DNA methylation participates SPATS2 activation in HCC. Therefore, the inhibition of the SNHG5/SPATS2 axis may be a potential strategy to increase the efficacy of sorafenib. Our findings can provide a reference for the targeted therapy of drug-resistant liver cancer.

In conclusion, we characterized SNHG5-DNMT3a-SPATS2 axis which is a novel oncogene combination that promotes the tumorigenesis and progression of HCC. We confirmed that the expression of SPATS2 may be regulated by SNHG5 via preventing DNMT3a-mediated the methylation of SPATS2. This study provides evidence that SNHG5 acts as an important factor for DNA methylation through the regulation of DNMT3 expression to expand the regulatory mechanism of SPATS2 targets. These findings may improve the understanding of the pathogenesis of HCC, and could provide a potential treatment strategy for liver cancer.

## Supporting information

S1 Dataset (XLSX)

S1 File (ZIP)

## References

[1] Siegel RL, Miller KD, Jemal A. Cancer statistics, 2019. CA Cancer J Clin. 2019; 0:1–28. doi: 10.3322/caac.21551 30620402

[2] Chen W, Zheng R, Baade PD, Zhang S, Zeng H, Bray F, et al. Cancer statistics in China, 2015. CA Cancer J Clin. 2016; 66(2):115–32. doi: 10.3322/caac.21338 26808342

[3] Beudeker BJB, Boonstra A. Circulating biomarkers for early detection of hepatocellular carcinoma. Therap Adv Gastroenterol. 2020; 13:1756284820931734. doi: 10.1177/1756284820931734 eCollection 2020. 32647536 PMC7325534

[4] Senoo M, Hoshino S, Mochida N, Matsumura Y, Habu S. Identification of a novel protein p59(scr), which is expressed at specific stages of mouse spermatogenesis. Biochem Biophys Res Commun. 2020; 292(4):992–8. doi: 10.1006/bbrc.2002.6769 11944913

[5] Ngollo M, Lebert A, Daures M, Judes G, Rifai K, Dubois L, et al. Global analysis of H3K27me3 as an epigenetic marker in prostate cancer progression. BMC Cancer. 2017; 17(1):261. doi: 10.1186/s12885-017-3256-y 28403887 PMC5388998

[6] Xing Jin; Tian Yijun; Ji Wu; Wang Xinying. Comprehensive evaluation of SPATS2 expression and its prognostic potential in liver cancer. Medicine. 2020; 9(9):19230. doi: 10.1097/MD.0000000000019230 32118724 PMC7478581

[7] Dong G, Zhang S, Shen S, Sun L, Wang X, Wang H, et al. SPATS2, negatively regulated by miR-145-5p, promotes hepatocellular carcinoma progression through regulating cell cycle. Cell Death Disease. 2020; 11(10):837. doi: 10.1038/s41419-020-03039-y 33037180 PMC7547105

[8] Damas ND, Marcatti M, Come C. SNHG5 promotes colorectal cancer cell survival by counteracting STAU1-mediated mRNA destabilization. Nature Communication. 2016; 7:13875. doi: 10.1038/ncomms13875 28004750 PMC5192221

[9] Li YH, Hu YQ, Wang SC, Li Y, Chen DM. LncRNA SNHG5: A new budding star in human cancers. Gene. 2020; 749:144724. doi: 10.1016/j.gene.2020.144724 32360843

[10] Li Y, Guo D, Zhao Y, Ren M, Lu G, Wang Y, et al. Long noncoding RNA SNHG5 promotes human hepatocellular carcinoma progression by regulating miR26a-5p/GSK3 beta signal pathway. Cell Death Disease. 2019; 9(9): 888. doi: 10.1038/s41419-018-0882-5 30166525 PMC6117363

[11] Li W, Lu Y, Wu Y, Qin Z, Tang Q, Wei H, et al. SNHG5 functions as competitive RNA with miR-23c to regulate HMGB2 expression in hepatocellular carcinoma. Am J Transl Research. 2020; 12(5): 2192–2200. 32509211 PMC7270010

[12] Hu PA, Miao YY, Yu S, Guo N. Long non-coding RNA SNHG5 promotes human hepatocellular carcinoma progression by regulating miR-363-3p/RNF38 axis. Eur Rev Med Pharmacol Sci. 2020; 24(7): 3592–3604. doi: 10.26355/eurrev_202004_20821 32329834

[13] Fernández-Barrena MG, Arechederra M, Colyn L, Berasain C, Avila MA. Epigenetics in hepatocellular carcinoma development and therapy: The tip of the iceberg. JHEP Report. 2020; 2(6):100167. doi: 10.1016/j.jhepr.2020.100167 33134907 PMC7585149

[14] Liu Y, Liu K, Tang C, Shi Z, Jing K, Zheng J. Long non-coding RNA XIST contributes to osteoarthritis progression via miR-149-5p/DNMT3A axis. Biomed Pharmacother. 2020; 128: 110349. doi: 10.1016/j.biopha.2020.110349 32521454

[15] Choudhari R, Sedano MJ, Harrison AL, Subramani R, Lin KY, Ramos EI, et al. Long noncoding RNAs in cancer: From discovery to therapeutic targets. Adv Clin Chemistry. 2020; 95:105–147. doi: 10.1016/bs.acc.2019.08.003 32122521

[16] Zhao Z, Sun W, Guo Z, Zhang J, Yu H, Liu B. Mechanisms of lncRNA/microRNA interactions in angiogenesis. Life Science. 2020; 254:116900. doi: 10.1016/j.lfs.2019.116900 31786194

[17] Li Y, Luo Q, Li Z, Wang Y, Zhu C, Li T, et al. Long Non-coding RNA IRAIN Inhibits VEGFA Expression via Enhancing Its DNA Methylation Leading to Tumor Suppression in Renal Carcinoma. Front Oncology. 2020; 10:1082. doi: 10.3389/fonc.2020.01082 32983957 PMC7492562

[18] Li X, Lv J, Liu S. MCM3AP-AS1 KD Inhibits Proliferation, Invasion, and Migration of PCa Cells via DNMT1/DNMT3 (A/B) Methylation-Mediated Upregulation of NPY1R. Mol Ther Nucleic Acids Research. 2020; 21:264–265. doi: 10.1016/j.omtn.2020.06.014 32610252 PMC7327873

[19] Zhang FF, Liu YH, Wang DW, Liu TS, Yang Y, Guo JM, et al. Obesity-induced reduced expression of the lncRNA ROIT impairs insulin transcription by downregulation of Nkx6.1 methylation. Diabetologia. 2020; 63(4): 811–824. doi: 10.1007/s00125-020-05090-y 32008054

[20] Lin J, Shi Z, Yu Z, He Z. LncRNA HIF1A-AS2 positively affects the progression and EMT formation of colorectal cancer through regulating miR-129-5p and DNMT3A. Biomed Pharmacother. 2018; 98: 433–439. doi: 10.1016/j.biopha.2017.12.058 29278853

[21] Xie Z, Wu Y, Liu S, Lai Y, Tang S. LncRNA-SNHG7/miR-29b/DNMT3A axis affects activation, autophagy, and proliferation of hepatic stellate cells in liver fibrosis. Clin Res Hepatol Gastroenterol. 2020; 4:101469. doi: 10.1016/j.clinre.2020.05.017 32893175

[22] Nagaraju GP, Dariya B, Kasa P, Peela S, El-Rayes BF. Epigenetics in hepatocellular carcinoma. Semin Cancer Biol. 2021: S1044-579X(21)00211-X. doi: 10.1016/j.semcancer.2021.07.017 34324953

[23] Lin JC, Yang PM, Liu TP. PERK/ATF4-Dependent ZFAS1 Upregulation Is Associated with Sorafenib Resistance in Hepatocellular Carcinoma Cells. Int J Mol Sci. 2021;22(11):5848. doi: 10.3390/ijms22115848 34072570 PMC8199104

