# Supplementary figures and images for: SPATS2 is positively activated by long noncoding RNA SNHG5 via regulating DNMT3a expression to promote hepatocellular carcinoma progression
Source: PLoS One. 2022 Jan 25;17(1):e0262262. doi: 10.1371/journal.pone.0262262 (PMC8789170; doi:10.1371/journal.pone.0262262)

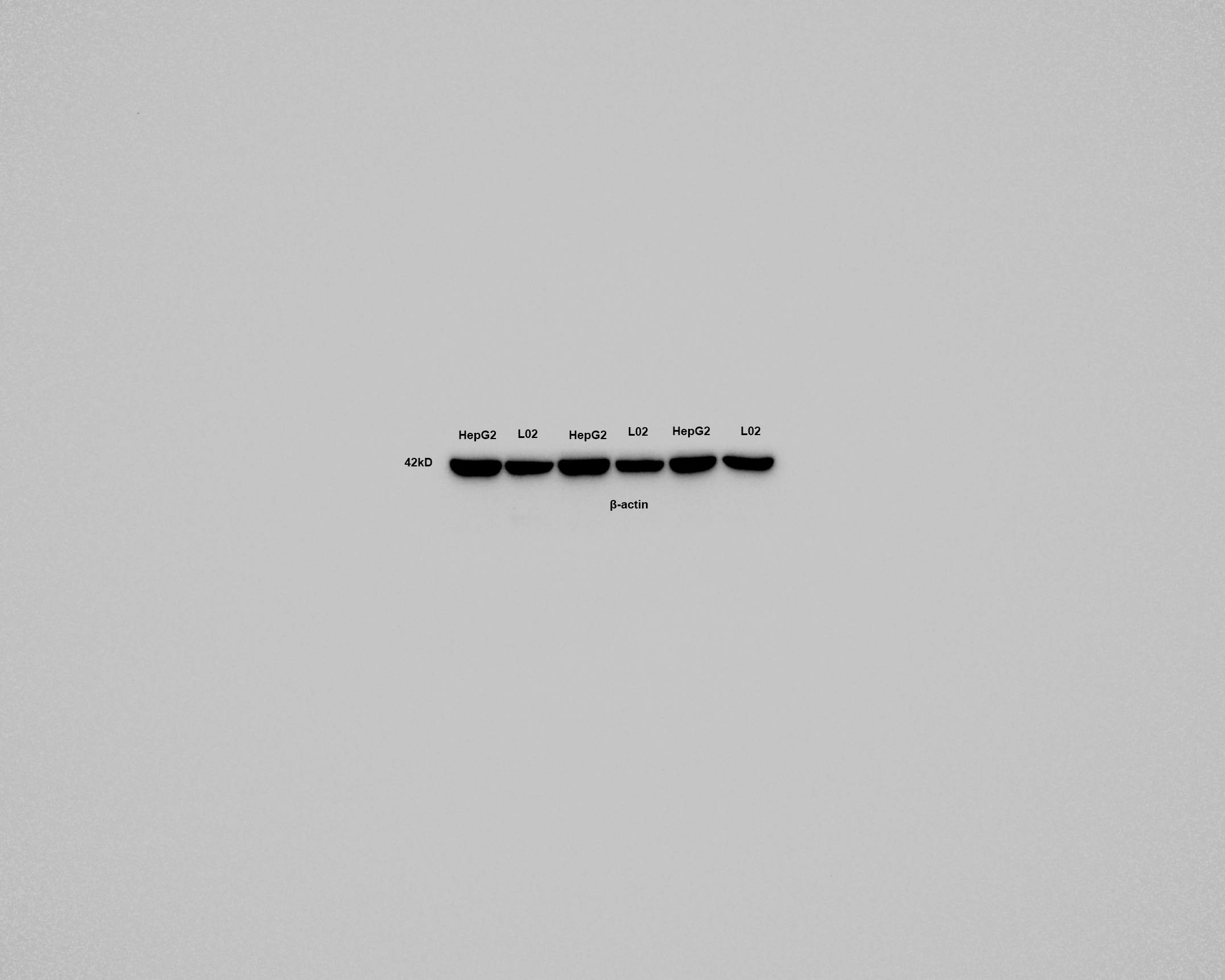

Supplement: S1 File — (ZIP) [file pone.0262262.s002.zip › western results/Figure 1D bate.tif]

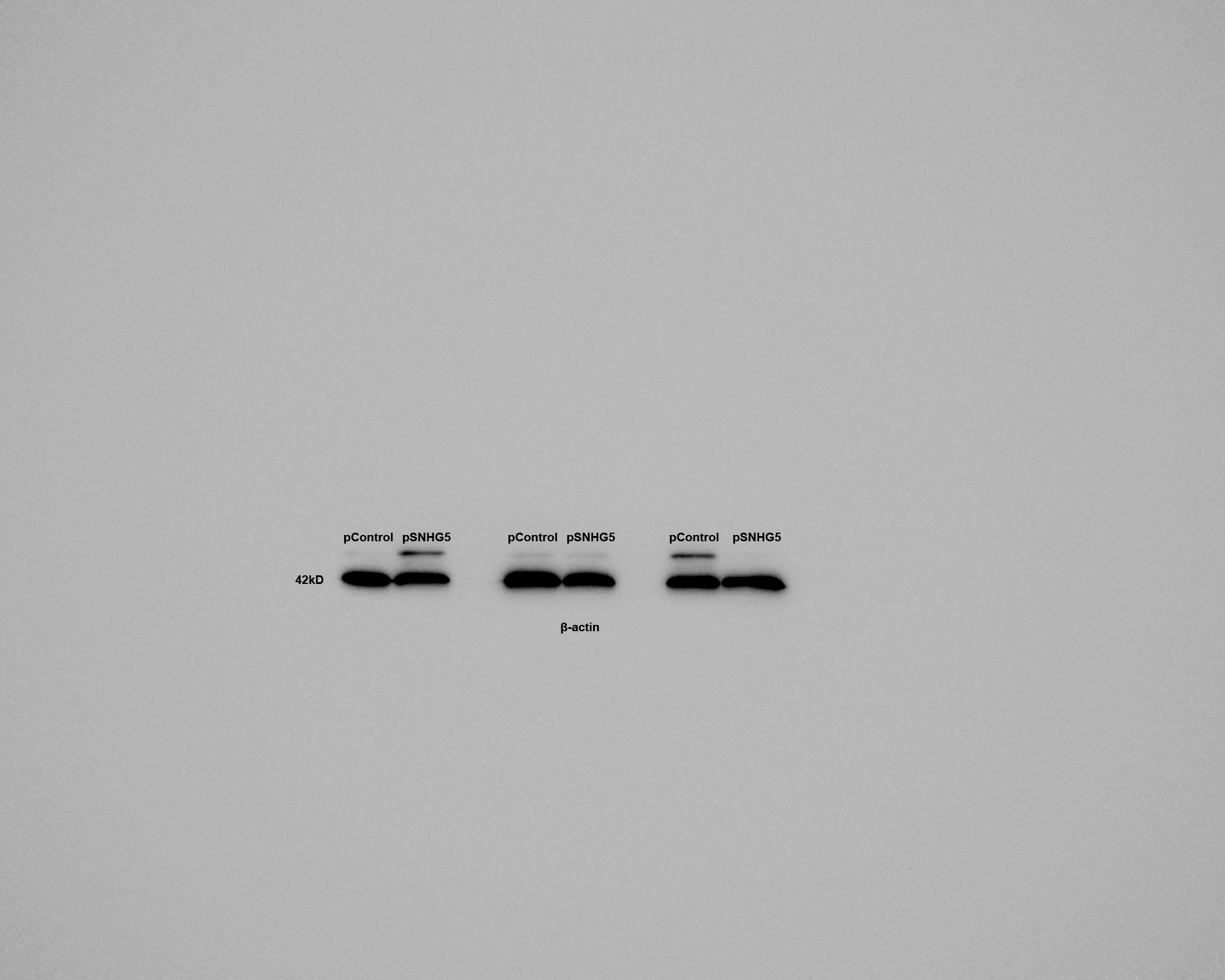

Supplement: S1 File — (ZIP) [file pone.0262262.s002.zip › western results/Figure 2D pSNHG5 bate.tif]

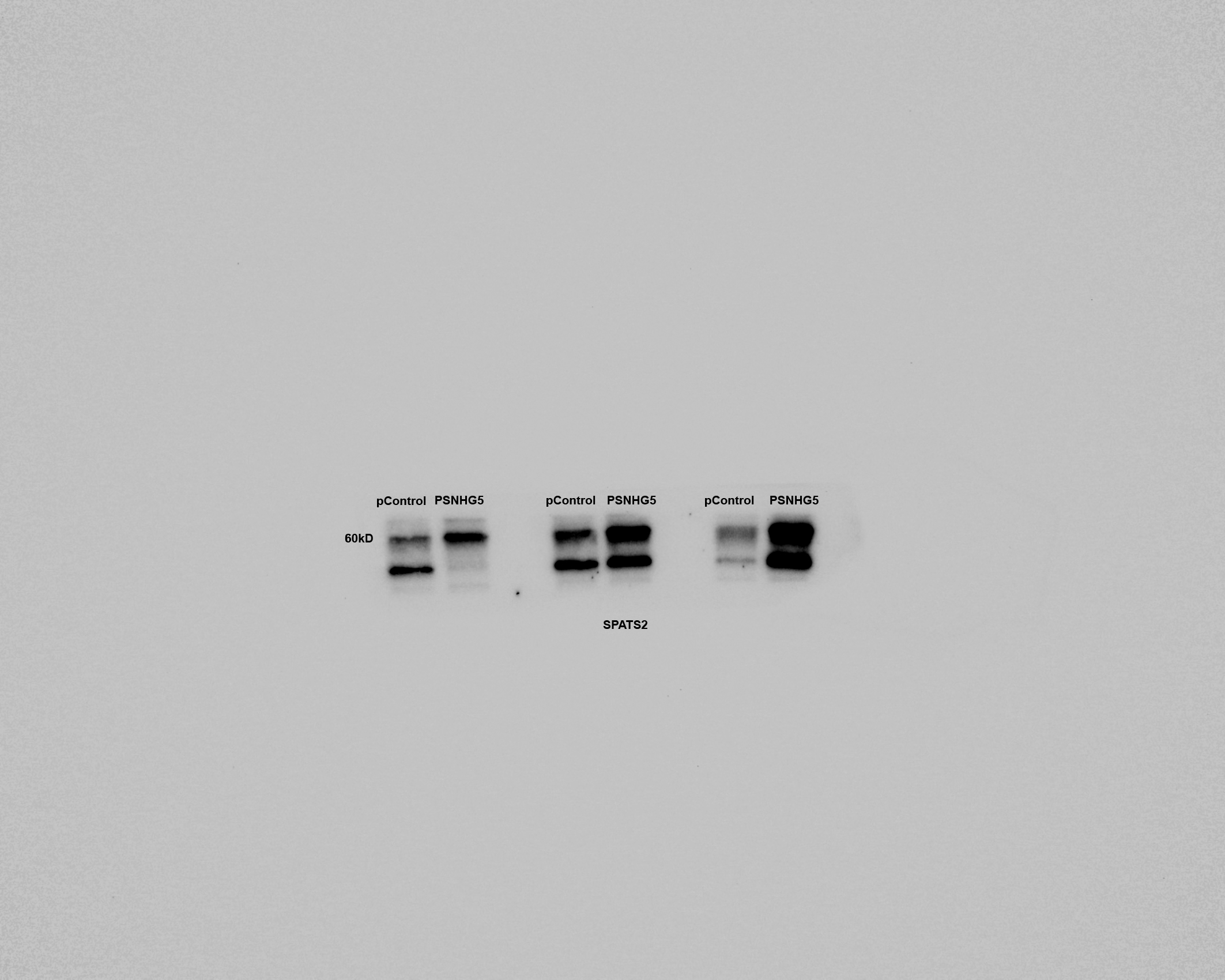

Supplement: S1 File — (ZIP) [file pone.0262262.s002.zip › western results/Figure 2D pSNHG5 spats2.tif]

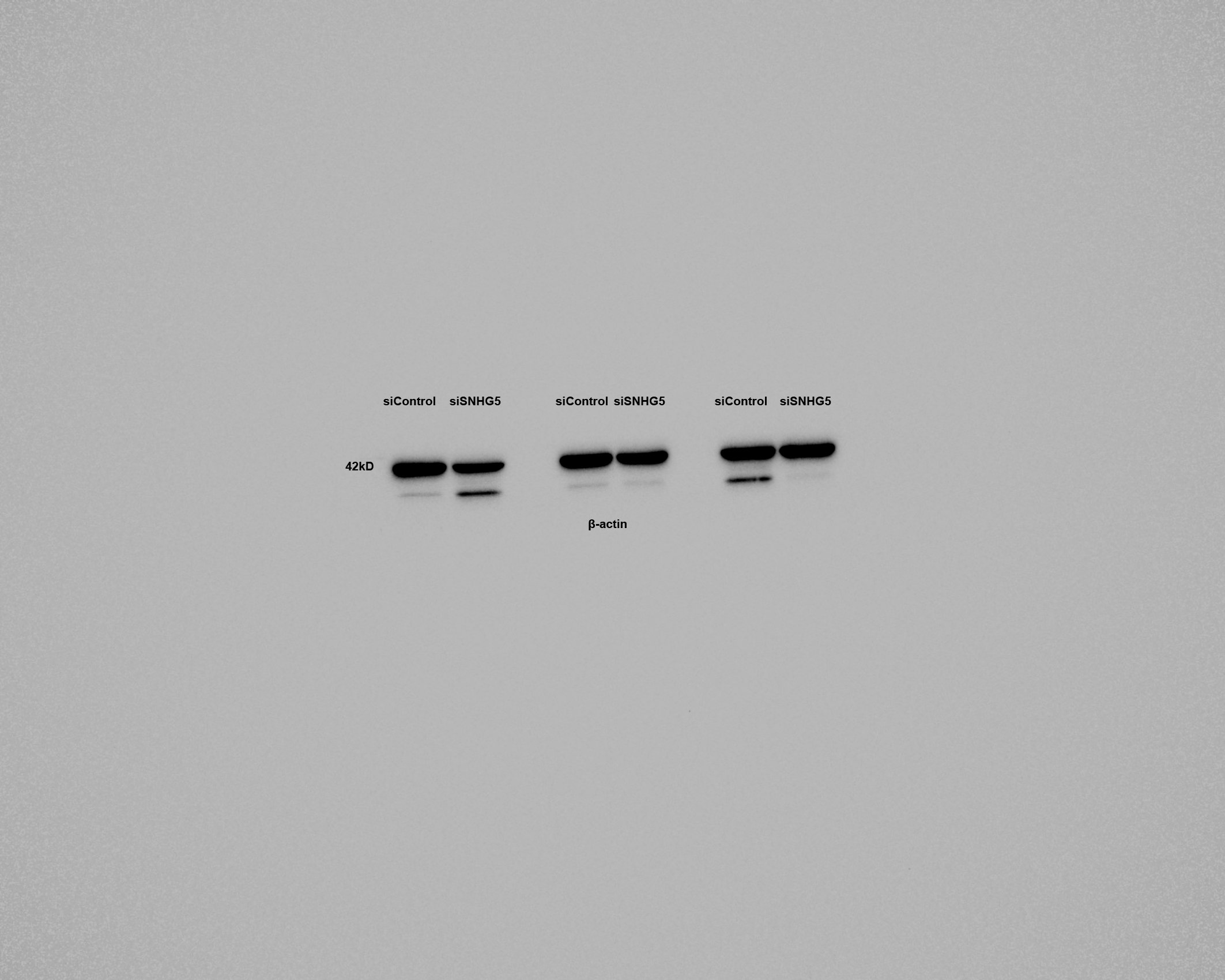

Supplement: S1 File — (ZIP) [file pone.0262262.s002.zip › western results/Figure 2D siSNHG5 bate.tif]

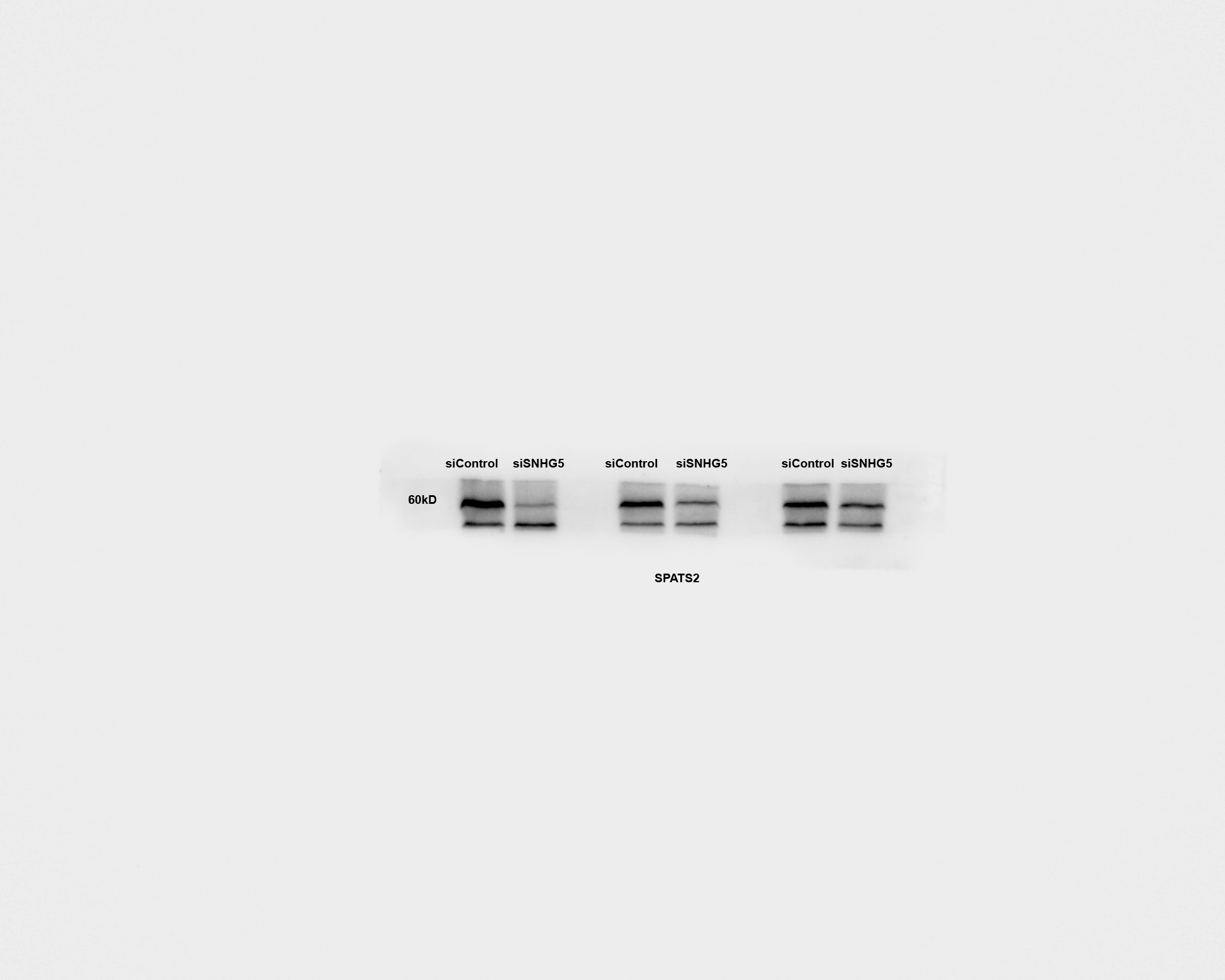

Supplement: S1 File — (ZIP) [file pone.0262262.s002.zip › western results/Figure 2D siSNHG5 SPATS2.tif]

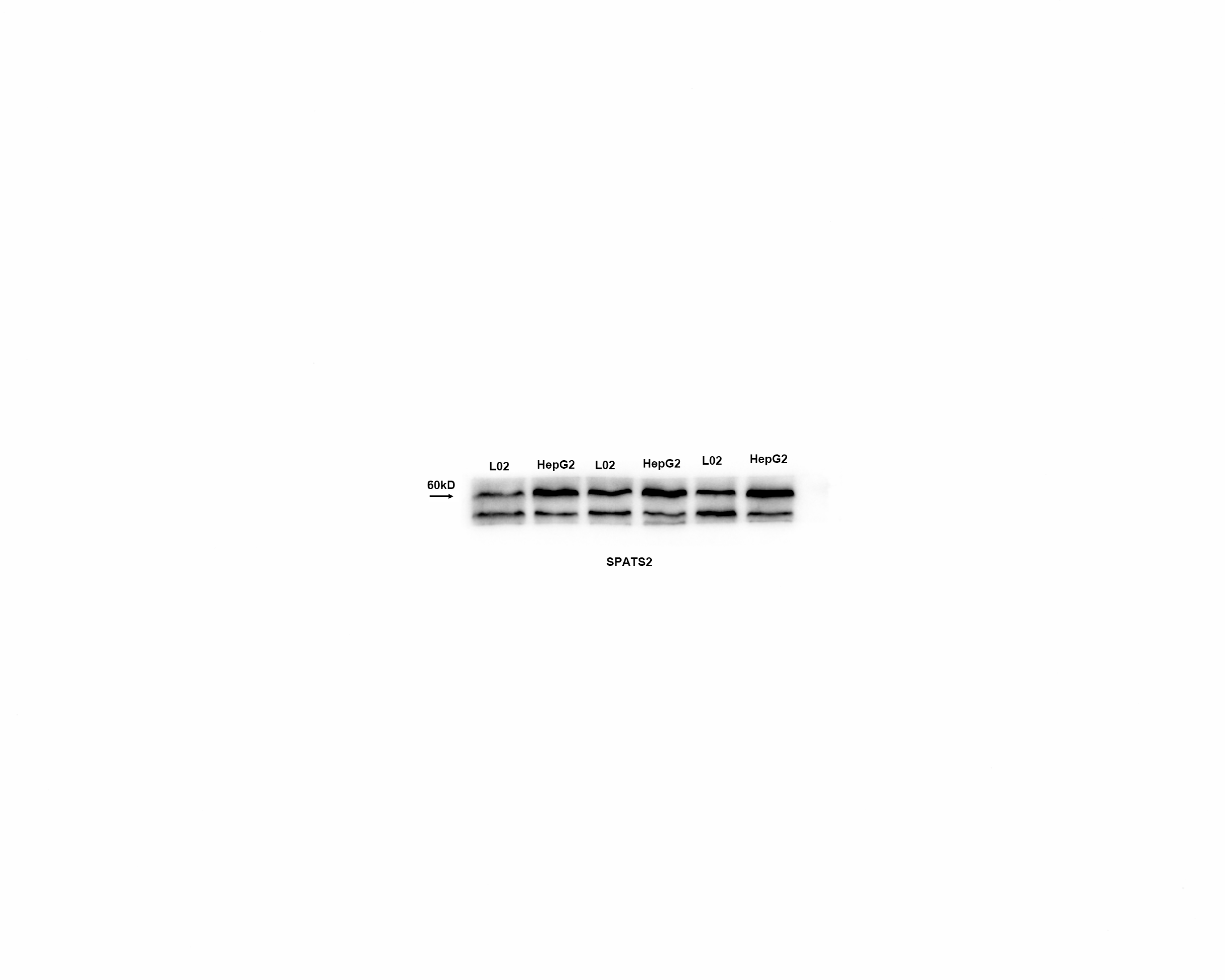

Supplement: S1 File — (ZIP) [file pone.0262262.s002.zip › western results/Figure1D SPATS2.tif]
